# Employment intentions of vocational nursing students toward geriatric care in central China

**DOI:** 10.3389/fpubh.2026.1871999

**Published:** 2026-09-21

**Authors:** Ting-ting Ruan, Ling-ling Huang, Li-juan Zhang

**Affiliations:** 1 Changsha Health Vocational College, Changsha, China; 2 Shanghai Donghai Vocational and Technical College, Shanghai, China; 3 The Third Xiangya Hospital of Central South University, Changsha, China

**Keywords:** employment intention, geriatric care, Social Cognitive Career Theory, vocational nursing students, workforce development

## Abstract

**Background:**

Global population aging has raised worldwide demand for geriatric care practitioners, including in China. Vocational nursing students are key reserve workforce for geriatric care, yet few enter this sector. Existing studies have explored individual factors shaping geriatric care employment intention, but few examine interactive effects of family support, experiential exposure and personal cognitive factors, or extend Social Cognitive Career Theory (SCCT) to Chinese vocational nursing education.

**Purpose:**

This study explores how family support, practical care experience and personal cognitive variables interact to shape vocational nursing students’ intentions to engage in geriatric care. It expands SCCT application and provides empirical evidence for geriatric care workforce recruitment, nursing curriculum design and may offer insights for other regions of China and aging societies internationally.

**Methods:**

A cross-sectional survey was conducted among 1,537 vocational nursing students in Hunan Province (2024–2025). Questionnaire data were analyzed using binary logistic regression and eXtreme Gradient Boosting (XGBoost) to identify key predictors. Hayes’ PROCESS macro was used to test the mediating role of personal factors.

**Results:**

Only 53.4% of students reported willingness to work in geriatric care. Family attitudes, prior caregiving experience and personal-factor-related occupational cognitions positively predicted intention, while insufficient curriculum exposure and poor perceived career prospects suppressed it. Personal factors significantly mediated the association between family factors and employment intention, with notable interactive effects across family, experience and cognition-related variables.

**Conclusion:**

Findings highlight integrating the family-experience-cognition pathway into curriculum optimization and policy interventions. This study enriches SCCT application in Chinese vocational education and offers region specific practical references for strengthening China’s geriatric care workforce via talent cultivation and educational reform.

## Background

The global aging population has generated a rapidly growing demand for geriatric care, creating a pressing workforce challenge worldwide (1). Despite policy initiatives such as wage increases and targeted immigration programs, many countries continue to experience severe shortages in long-term care (LTC). For example, Japan’s Ministry of Health, Labour and Welfare projects a deficit of approximately 570,000 caregivers by 2040, while German statistics anticipate a persistent gap between rising care needs and available staff (2, 3). China faces a similar challenge. According to the National Bureau of Statistics, 22.0% of the population was aged 60 or above in 2024, reflecting an increasing demand for professional LTC as family caregiving capacity weakens (4–6). However, most LTC workers in China have only junior middle school education or below, limiting service quality and older adults’ quality of life (7).

Although China graduates large cohorts of vocational nursing students each year, only 20–30% express an intention to work in geriatric care (8). This persistent mismatch underscores the need for evidence-based strategies to strengthen recruitment and retention in the field. Vocational nursing students represent a potentially significant pool to relieve China’s geriatric workforce shortage. In response, government ministries have issued guidelines to enhance higher vocational education in geriatric care and management (9). International comparisons reveal similar patterns: a global systematic review found approximately half of nursing students expressed willingness to care for older adults (10), while Korean and Italian studies also reported low geriatric intention among nursing students (11, 12). Moreover, educational interventions such as service learning have been shown to improve students’ attitudes and knowledge (13).

Further international evidence highlights the importance of pre-licensure interventions. In Turkey, a study of 688 nursing students revealed low intention to work in geriatric nursing, with professional values and contact experiences as significant predictors (14). In Italy, positive contact with older adults and higher academic year level increased willingness, while negative stereotypes reduced intention (12). A network meta-analysis further confirmed that pedagogical approaches—such as service learning, simulation, and community practicums—significantly improve knowledge, attitudes, and, in some cases, career intentions (15).

Despite these international advances, systematic empirical research on Chinese vocational nursing students remains limited. At the same time, nursing in China has become a nationally regulated major, with expanded undergraduate and postgraduate pathways and higher employment thresholds in hospitals. These structural shifts create both challenges and new opportunities for attracting vocational graduates to LTC (16–18). Existing evidence suggests that employment intention is shaped by a complex interplay of subjective career perceptions, family attitudes, curricular experiences, and policy awareness. However, relevant studies tend to explore these factors separately and lack systematic analysis of their interactive effects on career decision-making, particularly among Chinese vocational nursing students. To address this gap, this study introduces SCCT. Unlike single-dimension career theories, SCCT provides a holistic perspective to interpret the coordinated effects of environmental, experiential, and cognitive factors, which perfectly matches the multi-dimensional “family–experience–cognition” framework of this study.

According to SCCT, individual career intention is jointly shaped by three core components: contextual supports and barriers, experiential learning resources, and personal cognitive processes encompassing self-efficacy and outcome expectations (19). Contextual factors such as family attitudes provide proximal environmental resources or constraints for career decision-making, while experiential learning from caregiving practice and curriculum participation shapes individual occupational cognitions. As the most proximal predictors, personal cognitive processes directly drive career-related intentions. Guided by this theoretical logic, this study matches family factors to SCCT contextual conditions, care-related experience and curriculum exposure to experiential learning resources, and personal-factor-related occupational cognitions to self-efficacy and outcome expectations. This multi-dimensional framework was adopted to explore their combined effects and the cognitive mediating mechanism underlying vocational nursing students’ geriatric care employment intention.

To address this gap, this study surveyed 1,537 vocational nursing students in Hunan Province, central China, using logistic regression and eXtreme Gradient Boosting (XGBoost). Specifically, it seeks to answer the following questions:

(1) What factors shape vocational nursing students’ intentions to work in geriatric care?
(2) Does family support influence intention directly, or indirectly through personal factors related to occupational cognitions?
(3) Can the “family–experience–cognition” framework be empirically validated in the Chinese context?

Based on SCCT theoretical propositions and the above research questions, the following testable hypotheses were proposed:

*H1*: Family factors (family support and family occupational evaluation) are associated with vocational nursing students’ geriatric care employment intention.

*H2*: Practical experiential factors (prior caregiving experience, geriatric nursing curriculum exposure) positively predict students’ geriatric care employment intention.

*H3*: Personal-factor-related occupational cognitions exert a positive predictive effect on geriatric care employment intention.

*H4*: Personal-factor-related occupational cognitions play a mediating role in the relationship between family factors and geriatric care employment intention.

By addressing these questions and testing the above hypotheses, the study aims to generate evidence-based insights to guide workforce development and curriculum reform in geriatric care.

## Methods

### Participants and setting

A cross-sectional survey design was employed in this study to assess the employment intentions of vocational nursing students toward geriatric care and to explore the influencing factors. Participants were recruited from vocational colleges in Hunan Province, China, between November 2024 and January 2025 using convenience sampling. Participants were enrolled via two recruitment routes: regular-year students were accessed through college-level class communication channels, while third-year students on clinical rotation were invited by hospital preceptors via online questionnaire links. Sample-size consideration followed the 10-events-per-predictor rule for binary logistic regression, consistent with sample sizes adopted in comparable cross-sectional studies on nursing students’ geriatric care career intention. All returned questionnaires underwent standardized quality screening. Responses were excluded if completion time was less than 1 min (indicating careless responding), showed uniform ceiling/floor response patterns, or contained substantial incomplete data. Following these pre-specified exclusion criteria, 1,537 valid questionnaires were retained for final analysis. Inclusion criteria were: (1) full-time students enrolled in nursing programs at higher vocational institutions; (2) having studied nursing for more than 6 months; and (3) providing informed consent to participate voluntarily.

### Instruments

The survey instrument was a meticulously structured questionnaire, segmented into three main sections:

*Demographic Information*: This section aimed to collect basic demographic data set by the researchers, including gender, residence, only-child status, academic year, prior caregiving experience, duration of co-residence with older adults, and geriatric nursing exposure (defined as whether participants have studied geriatric nursing-related courses or read relevant books), parental support (i.e., parental attitudes toward working in the geriatric care sector), entrepreneurial intention, and annual household income.

*Employment Intention Questionnaire*: This section was designed to assess vocational nursing students’ willingness to pursue a career in geriatric care. It included items covering overall employment willingness, perceived occupational barriers, motivating factors, institutional and job preferences, and salary expectations. The items were adapted from the “*Questionnaire on Chinese College Students’ Employment Intentions in the Older Adult Care Industry*” developed by the China Philanthropy Research Institute at Beijing Normal University (20).

*Influencing Factors Questionnaire*: This Chinese questionnaire was adapted from Zhu (21), originally developed for students majoring in older adult care services. For our sample of vocational nursing students, we adapted the profession-specific wording to ‘geriatric care’ while keeping the original 4 primary dimensions and 14 secondary indicators:

(1) Personal Factors: Including interest, expectations, abilities, and beliefs, consisting of 14 items scored on a 5-point scale (1 = strongly disagree, 2 = disagree, 3 = neutral, 4 = agree, 5 = strongly agree).
(2) External Environmental Factors: Covering national and local policies, urban infrastructure, societal attitudes, and school education, consisting of 15 items scored on a 5-point scale (1 = very unimportant, 2 = unimportant, 3 = neutral, 4 = important, 5 = very important).
(3) Workplace Factors: Encompassing salary, facilities, and organizational culture, consisting of 10 items scored on a 5-point scale (1 = very unimportant, 2 = unimportant, 3 = neutral, 4 = important, 5 = very important).
(4) Family Factors: Involving family support, occupational views, and family expectations, consisting of 10 items scored on a 5-point scale (1 = very unimportant, 2 = unimportant, 3 = neutral, 4 = important, 5 = very important).

This questionnaire has shown good reliability and validity, indicating its suitability for the purposes of this study.

### Data collection

Data were collected electronically using an online questionnaire platform. The survey link was distributed via official class communication channels (e.g., WeChat groups and school internal networks). Participants completed the survey anonymously and voluntarily. Duplicate and incomplete responses were removed prior to analysis.

### Statistical analysis

Descriptive statistics (frequencies and percentages) were used to summarize participants’ demographic characteristics and geriatric care employment intention. Cronbach’s *α*, exploratory factor analysis (EFA) and confirmatory factor analysis (CFA) were conducted to test the reliability and construct validity of the questionnaire. Convergent and discriminant validity were further evaluated using average variance extracted (AVE), composite reliability (CR) and the Fornell-Larcker criterion.

Chi-square tests were applied to examine bivariate associations between each demographic variable and geriatric care employment intention, and were not used as a variable-selection criterion for the multivariate model. Saturated binary logistic regression with backward elimination was performed to identify independent predictors of geriatric care employment intention (0 = unwilling, 1 = willing). Model fit, multicollinearity and predictive performance were assessed, and odds ratios (OR) with 95% confidence intervals were calculated.

An XGBoost model was constructed for cross-validation of prediction performance. The dataset was randomly split into an 80% training set and a 20% independent test set. Grid-search hyperparameter tuning was implemented with 5-fold stratified cross-validation. Feature importance was extracted for model interpretation.

Hayes’ PROCESS macro 4.1 (Model 4) was used to examine the mediating role of personal factors. Given the binary outcome, logit-link logistic mediation with 5,000 percentile Bootstrap resamples was adopted. Residual diagnostics were performed for the first-stage ordinary least-squares regression. All tests were two-tailed with significance set at *p* < 0.05. Analyzes including descriptive statistics, chi-square tests, logistic regression and mediation analysis were conducted using SPSS 26.0. XGBoost modeling was implemented in Python.

## Results

### Overall employment intention in geriatric care

This study recruited 1,537 vocational nursing students from Hunan Province, China. The results showed that only 53.4% of the students expressed a willingness to engage in geriatric care. Among those not willing to pursue a career in geriatric care, the primary barriers identified were “low salary” (61.5%), “strenuous working conditions” (49.1%), and “difficulty in communicating with older adults” (47.8%). More than 80% of the students indicated that policy incentives, such as employment subsidies and social security subsidies, could improve their employment intentions. “National policy orientation” was cited by 61.3% of the students as an important factor encouraging them to consider entering the geriatric care industry. “Broad industry prospects” (64.3%) and “less social pressure” (52%) were also significant motivations for employment.

With respect to employment preferences, public nursing homes (72.2%), hospitals (62.5%), and private nursing homes (61.2%) were the most frequently selected settings, reflecting a strong reliance on the medical system. Among job types, health management (72.6%) and nutrition catering (65.6%) attracted the most interest, whereas routine daily care (37.2%) and end-of-life care (28%) were chosen by considerably fewer students. The most commonly expected pre-tax monthly salary ranges were 5,001–7,000 CNY (35.7%) and 7,001–9,000 CNY (24.1%), both above the current industry average, indicating a gap between salary expectations and reality.

### Demographic factors affecting employment intention

Univariate associations between independent variables and vocational nursing students’ willingness to engage in geriatric care were examined using chi-square tests. Gender, residence, academic year, prior caregiving experience, geriatric nursing exposure, parental support, and entrepreneurial intention were significantly associated with geriatric care employment intention (*p* < 0.05). Duration of co-residence with older adults showed a marginally significant association with employment intention (*p* = 0.050). In contrast, only-child status and annual household income exhibited no significant between-group differences (*p* > 0.05). Full details are provided in Table 1.

**Table 1:** Demographic differences in vocational nursing students’ intention to work in geriatric care.

Variable	Category	Willing to work in geriatric care		*χ* ^2^	*p*
		Yes (%)	No (%)		
Gender	Male	79 (63.7)	45 (36.3)	5.816	0.016
	Female	741 (52.4)	672 (47.6)		
Residence	Rural	646 (54.7)	534 (45.3)	3.973	0.046
	Urban	174 (48.7)	183 (51.3)		
Academic year	Year 1	456 (51.6)	427 (48.4)	7.298	0.026
	Year 2	201 (52.1)	185 (47.9)		
	Year 3	163 (60.8)	105 (39.2)		
Prior caregiving experience	Yes	489 (64.5)	269 (35.5)	74.859	0.000
	No	331 (42.5)	448 (57.5)		
Geriatric nursing exposure	Yes	333 (64.2)	186 (35.8)	36.800	0.000
	No	487 (47.8)	531 (52.2)		
Parental support	Supportive	406 (75.7)	130 (24.3)	172.553	0.000
	Opposed	32 (29.6)	76 (70.4)		
	Neutral	382 (42.8)	511 (57.2)		
Entrepreneurial intention	Yes	336 (75.3)	110 (24.7)	122.033	0.000
	No	484 (44.4)	607 (55.6)		
Annual household income (CNY)	<30 k	410 (55.9)	324 (44.1)	6.338	0.175
	30–60 k	212 (52.0)	196 (48.0)		
	60–100 k	110 (50.9)	106 (49.1)		
	100–150 k	60 (51.7)	56 (48.3)		
	>150 k	27 (41.5)	38 (58.5)		
Only child	No	707 (54.1)	599 (45.9)	2.147	0.143
	Yes	113 (48.9)	118 (51.1)		
Duration of co-residence with older adults	0	168 (47.3)	187 (52.7)	11.095	0.050
	<6 months	68 (47.9)	74 (52.1)		
	6–12 months	35 (53.8)	30 (46.2)		
	1–2 years	50 (53.2)	44 (46.8)		
	2–3 years	35 (53.8)	31 (46.2)		
	≥3 years	464 (56.9)	351 (43.1)		

Percentages denote within-group (row-level) proportions for categorical and ordinal variables. Chi-square tests were applied to compare geriatric care employment intention across subgroups. Annual household income and duration of co-residence with older adults are treated as ordinal categorical variables in this table.

### Assessment of influencing factors

#### Reliability and validity of the questionnaire

The adapted Chinese scale Questionnaire on Factors Influencing Employment in Geriatric Care was originally developed by Zhu (21) for students majoring in older adult care services. Minor wording revisions were performed to adapt the scale to vocational nursing students. Given the differences between the original and current study populations, exploratory factor analysis (EFA) was first conducted to examine the factor structure. Confirmatory factor analysis (CFA) was then used to test the hypothesized four-dimensional theoretical framework.

The reliability of the questionnaire was assessed using Cronbach’s *α*, yielding an overall coefficient of 0.988. This coefficient indicates excellent internal consistency for the instrument as a whole. The *α* coefficients for the four dimensions-external environmental factors (0.983), personal factors (0.986), family factors (0.975), and workplace factors (0.983)-all exceeded 0.9, further supporting the instrument’s strong reliability.

In the validity analysis, EFA extracted four common factors that accounted for 83.97% of the total variance, consistent with the dimensional structure of the original scale. CFA provided further support for the construct validity of the four-factor model. Standardized factor loadings for all items ranged from 0.721 to 0.957 (*p* < 0.001). All dimensions demonstrated satisfactory convergent validity, with AVE values ranging from 0.798 to 0.873 and CR values ranging from 0.975 to 0.986. The Fornell–Larcker criterion indicated acceptable discriminant validity among the four dimensions.

#### Key predictors of employment intention

Binary logistic regression was used to identify independent predictors of vocational nursing students’ willingness to engage in geriatric care. The dependent variable was dichotomous as 0 = unwilling to work in geriatric care and 1 = willing. The independent variables comprised all demographic covariates. To avoid omitted-variable bias, each demographic indicator was retained in the saturated full model irrespective of univariate significance; non-significant predictors were then removed by backward elimination after adjustment for all other covariates. The model fitted the data acceptably, with likelihood-ratio *χ*^2^ = 381.222 (*p* < 0.001) and Hosmer–Lemeshow goodness-of-fit *p* = 0.765. The pseudo-*R*^2^ values were 0.179 (McFadden) and 0.293 (Nagelkerke). The overall classification accuracy reached 69.94%. All variance inflation factor (VIF) values were below 5, indicating no serious multicollinearity. Detailed regression outputs are summarized in Table 2; odds ratios (OR) with corresponding 95% confidence intervals are shown in Figure 1.

**Table 2:** Summary of binary logit regression results.

Variable	Group	*β*	SE	*z*	Wald *χ*^2^	*p*	OR	95% CI	VIF
Entrepreneurial intention (VS: No)	Yes	1.046	0.138	7.561	57.164	0.000	2.847	2.171–3.734	1.112
Parental support (VS: Neutral)	Opposed	−0.497	0.241	−2.060	4.244	0.039	0.608	0.379–0.976	1.053
	Supportive	1.221	0.131	9.325	86.958	0.000	3.390	2.623–4.381	1.127
Geriatric nursing exposure (VS: No)	Yes	0.293	0.128	2.286	5.224	0.022	1.340	1.043–1.723	1.085
Prior caregiving experience (VS: No)	Yes	0.586	0.119	4.929	24.300	0.000	1.796	1.423–2.268	1.091
Annual household income		−0.129	0.052	−2.483	6.168	0.013	0.879	0.794–0.973	1.008
Workplace factors		0.044	0.012	3.777	14.269	0.000	1.045	1.021–1.069	2.410
Family factors		−0.080	0.014	−5.575	31.086	0.000	0.924	0.898–0.950	3.419
Personal factors		0.048	0.007	6.630	43.962	0.000	1.049	1.034–1.064	2.019
Intercept		−1.798	0.362	−4.972	24.721	0.000	0.166	0.082–0.337	
Likelihood ratio test	*χ*^2^ (9) = 381.222, *p* = 0.000								
Hosmer–Lemeshow test	*χ*^2^ (8) = 4.931, *p* = 0.765								
McFadden *R*^2^ = 0.179 Nagelkerke *R^2^ = 0.293*									

**Figure 1:**
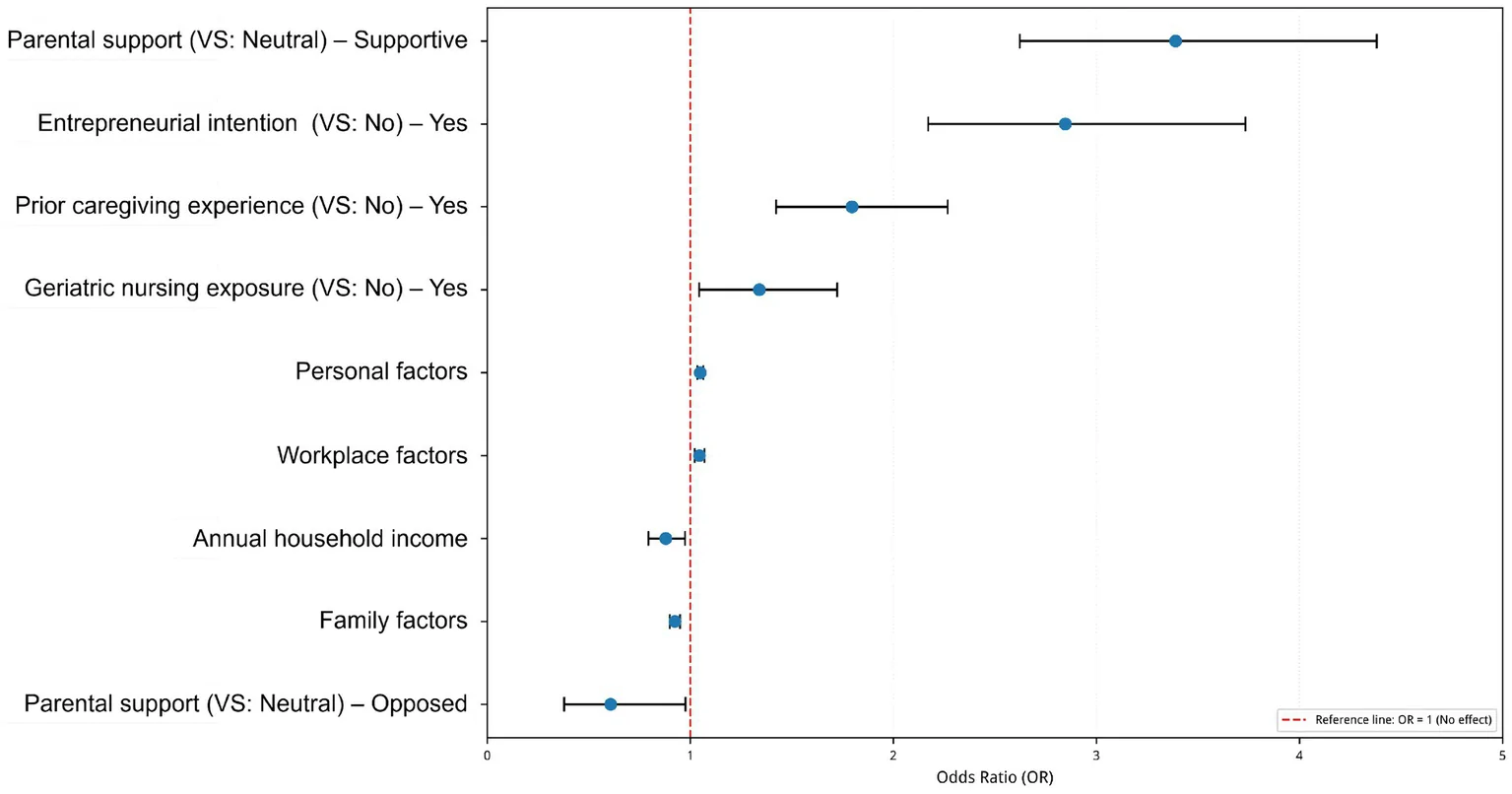
Dot-and-whisker plot of odds ratios and 95% confidence intervals.

Perceived supportive parental attitudes had the strongest positive association with employment intention (OR = 3.390, *p* < 0.001). Entrepreneurial intention (OR = 2.847, *p* < 0.001) and prior caregiving experience (OR = 1.796, *p* < 0.001) were also associated with greater willingness to enter geriatric care, whereas geriatric-nursing exposure showed a weaker positive association (OR = 1.340, *p* = 0.022). After confounder adjustment, personal factors (OR = 1.049, *p* < 0.001) and workplace factors (OR = 1.045, *p* < 0.001) had weak but statistically significant positive associations. By contrast, the composite family-factors scale (OR = 0.924, *p* < 0.001) and annual household income were inversely associated with employment intention. Two variables that were significant in bivariate chi-square testing were excluded from the final regression equation. Academic year was significant in univariate analysis (*χ*^2^ = 7.298, *p* = 0.026) but lost independent predictive power after adjustment for experiential and attitudinal covariates; uneven distribution across grade groups further attenuated its statistical power. Only-child status, though culturally relevant in the Chinese context, showed no significant association with willingness to enter geriatric care in either univariate chi-square testing (*χ*^2^ = 2.147, *p* = 0.143) or the saturated fully adjusted regression model.

The model’s overall prediction accuracy was 69.94%, and the corresponding accuracy among those willing to work in geriatric care was 71.34% (see Table 3 for details). The ROC curve in Figure 2, with an AUC of 0.775 (95% CI: 0.752–0.798), nonetheless indicates satisfactory discriminatory performance.

**Table 3:** Classification accuracy of the binary logistic regression model.

Observed outcome		Predicted outcome		Accuracy (%)	Error rate (%)
		0	1		
Observed	0	490	227	68.34%	31.66%
	1	235	585	71.34%	28.66%
Overall				69.94%	30.06%

**Figure 2:**
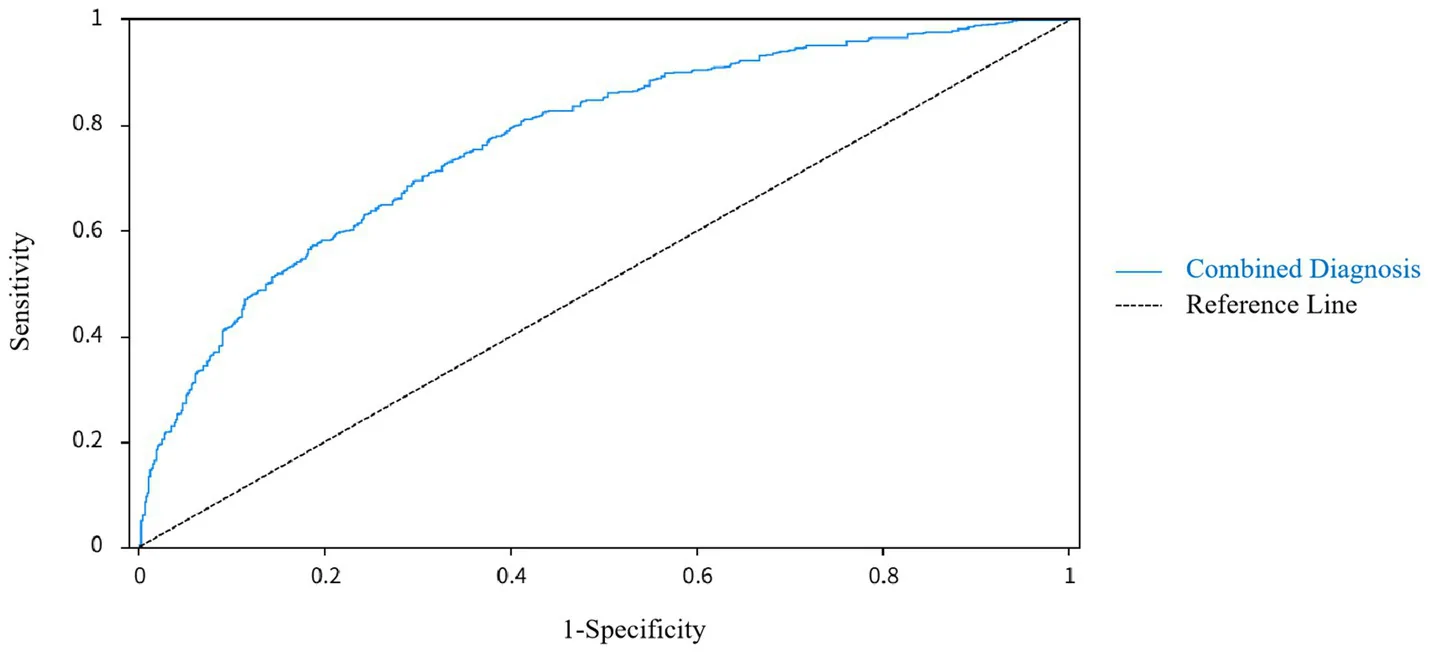
ROC curve for combined diagnosis model.

To assess the predictive performance of the logistic regression model, an XGBoost model was constructed using identical independent and dependent variables as those adopted in the binary logistic regression. Hyperparameters were globally optimized via grid search combined with 5-fold stratified cross-validation. A random seed of 42 was set, and the dataset was split at an 8:2 ratio into a training set (1,229 participants) and an independent test set (308 participants). After evaluating 65,536 hyperparameter combinations, the optimal configuration was determined as follows: colsample_bytree = 0.7, gamma = 0.3, learning rate = 0.1, max_depth = 2, n_estimators = 100, L1 regularization = 1.0, L2 regularization = 2.0, and subsample = 0.7. The model achieved a 5-fold cross-validation AUC of 0.7609, an independent test-set AUC of 0.8186, and a test-set accuracy of 77.92%. The confusion matrix on the test set comprised 106 true negatives, 38 false positives, 30 false negatives, and 134 true positives. The corresponding ROC curve is presented in Figure 3.

**Figure 3:**
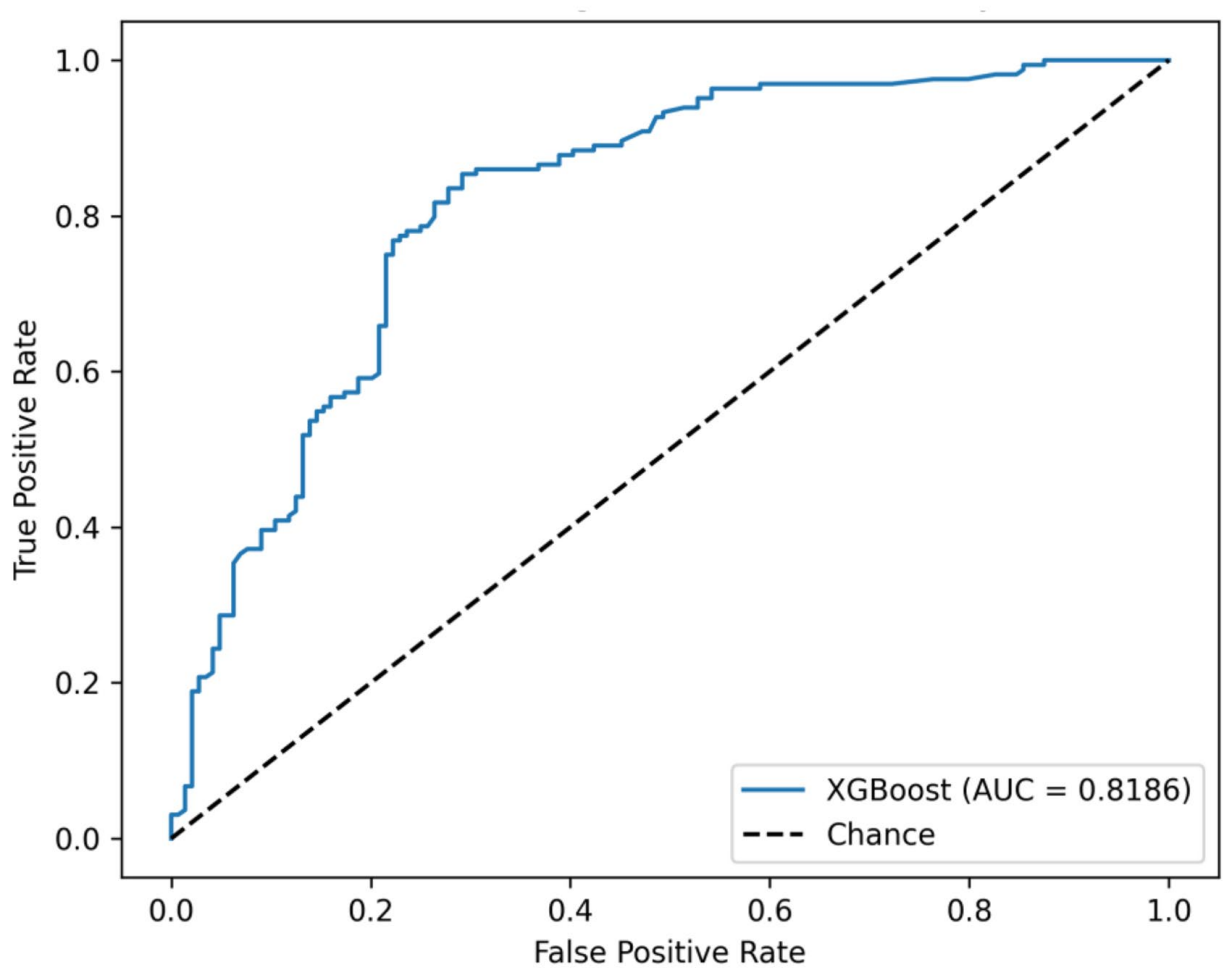
XGBoost ROC curve for geriatric care employment intention (test set).

XGBoost gain-based feature importance scores were sorted in descending order: parental support-supportive (0.3271), entrepreneurial intention (0.1636), and prior caregiving experience (0.1596). Full feature-importance results are tabulated and visualized as a horizontal bar chart in Figure 4, and the model showed good discriminative capacity for the binary outcome.

**Figure 4:**
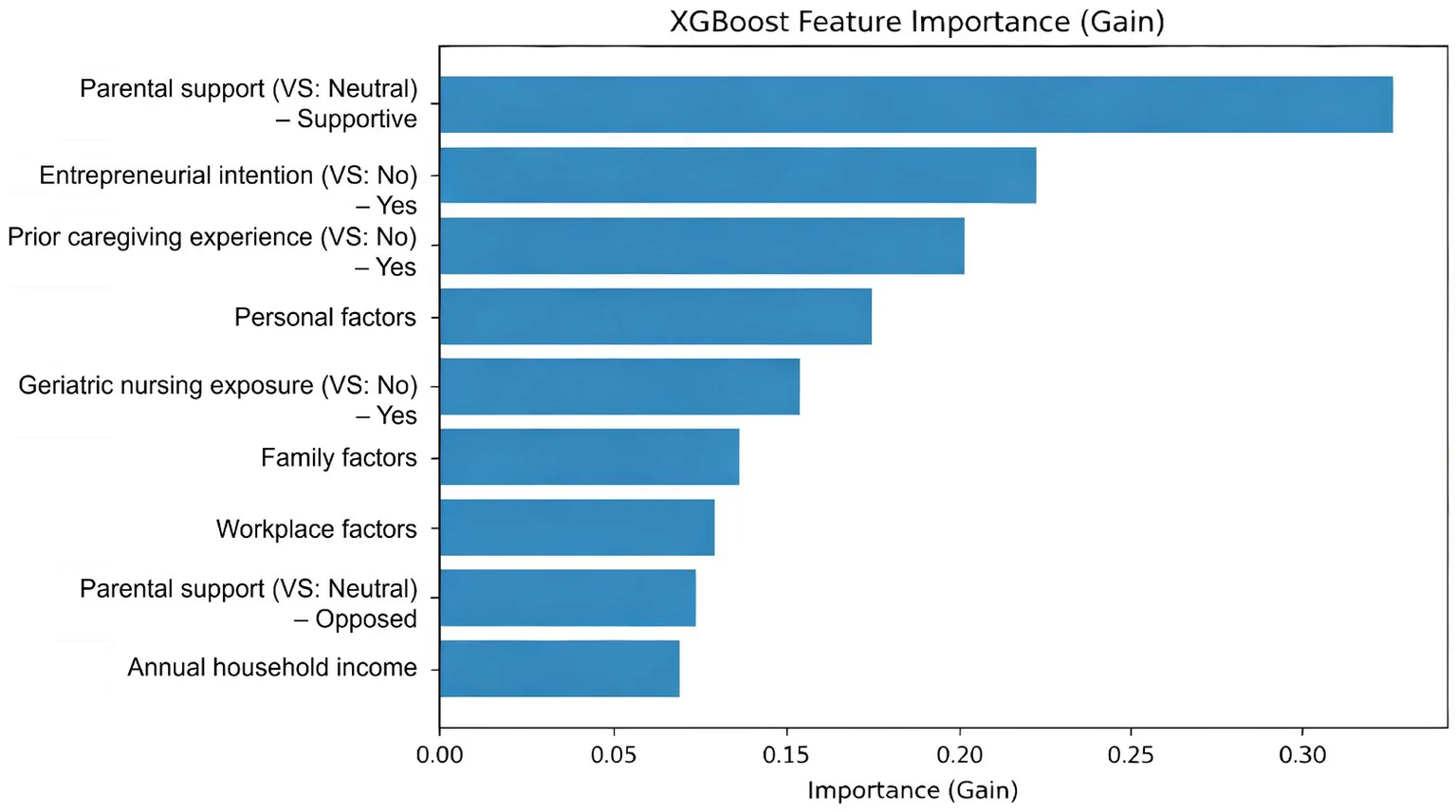
Feature importance horizontal bar plot of optimized XGBoost model.

#### Mediation analysis of influencing factors

Binary logistic regression demonstrated that scores for workplace factors, personal factors, and family factors independently predicted vocational nursing students’ geriatric care employment intention after adjusting for demographic confounders (Table 2), with the composite family factor scale exerting a negative predictive effect. To clarify the mechanism underlying this negative association, Hayes’ PROCESS macro Model 4 with 5,000 bootstrap resamples was used to test the mediating role of personal factors in the association between family factors and employment intention. Because the outcome variable was binary, the macro automatically implemented logistic-based mediation using a logit link function. Logistic mediation under a logit link operates on a non-linear log-odds scale, which violates the additive decomposition *c* = *c*′ + *ab* assumed in ordinary least-squares mediation; total-effect statistics are therefore not reported in Table 4.

**Table 4:** Mediation effect of personal factors on the relationship between family factors and intention to work in geriatric care.

Path	Variable	*β*	SE	Test statistic (t/Z)	*p*	95% CI
Step 1: *X* → *M* (DV: personal factors)	Constant	8.75	1.199	7.296	<0.001	[6.398, 11.103]
	Family factors (path *a*)	1.075	0.029	36.968	<0.001	[1.018, 1.133]
Model fit	–	*R*^2^ = 0.471, *F* = 1366.65, *p* < 0.001				
Step 2: *X* + *M* → *Y*	Constant	−1.27	0.281	−4.522	<0.001	[−1.820, −0.720]
(DV: employment intention, logistic)	Family factors (direct effect *c′*)	−0.044	0.010	−4.507	<0.001	[−0.064, −0.025]
	Personal factors (path *b*)	0.061	0.007	9.337	<0.001	[0.048, 0.074]
Model fit	–	−2LL = 2,012.49, Nagelkerke *R*^2^ = 0.093, *p* < 0.001				
Effect decomposition	Direct effect (*X* → *Y*, *c*′)	−0.044	0.010	−4.507	<0.001	[−0.064, −0.025]
	Indirect effect (*X* → *M* → *Y*, *a × b*)	0.066	0.007	–	–	[0.053, 0.079]

Bootstrap resamples = 5,000. CI = confidence interval. Logistic mediation with logit link was used for the binary dependent variable; nonlinear log-odds scale does not support additive decomposition of total, direct and indirect effects, so total effect values are not presented in this table.

The mediation model comprised two analytical stages. Stage 1 applied ordinary least-squares regression to predict the continuous mediator (personal factors) from family factors. Stage 2 fitted logistic regression to estimate path b (the predictive effect of personal factors) and the direct effect c′ of family factors on employment intention after controlling for the mediator. Family factors had a significant negative direct effect on employment intention (*c*′ = −0.044, SE = 0.010, *p* < 0.001). Path a showed that family factors significantly and positively predicted personal factors (*β* = 1.075, *p* < 0.001). Path b indicated that personal factors positively predicted geriatric-care employment intention (*β* = 0.061, *p* < 0.001).

In this mediation model, the direct effect of family factors was negative (*c*′ = −0.044), whereas the indirect effect transmitted through personal factors was significantly positive (*a* × *b* = 0.066). The unstandardized indirect effect of 0.066 could be regarded as a medium sized effect within the present study context. The ratio of the indirect effect to the unadjusted zero-order association exceeded 1, which is characteristic of a suppressor-effect pattern. This pattern indicates that the zero-order correlation between family factors and employment intention is dominated by the positive indirect pathway. After accounting for the mediating variable, the remaining direct effect mainly reflects household-level occupational barriers captured within the family-factors scale. In addition, the ten items of the family-factors dimension contain both facilitative and restrictive elements. Aggregation into a composite total score leads to the mutual offsetting of opposing item-level effects, which explains the seemingly paradoxical result of a negative direct coefficient paired with a positive indirect pathway. Due to the non-linear nature of logistic-based mediation, fully standardized indirect-effect coefficients cannot be generated because the variance of the binary outcome is not fixed on the log-odds scale; accordingly, only raw unstandardised path coefficients are presented throughout the manuscript (22). The conventional indirect/total ratio is not interpretable under this suppressor-effect pattern, and Kenny’s full/partial mediation labels are not appropriate for logistic mediation with opposite-sign direct and indirect effects (Figure 5).

**Figure 5:**
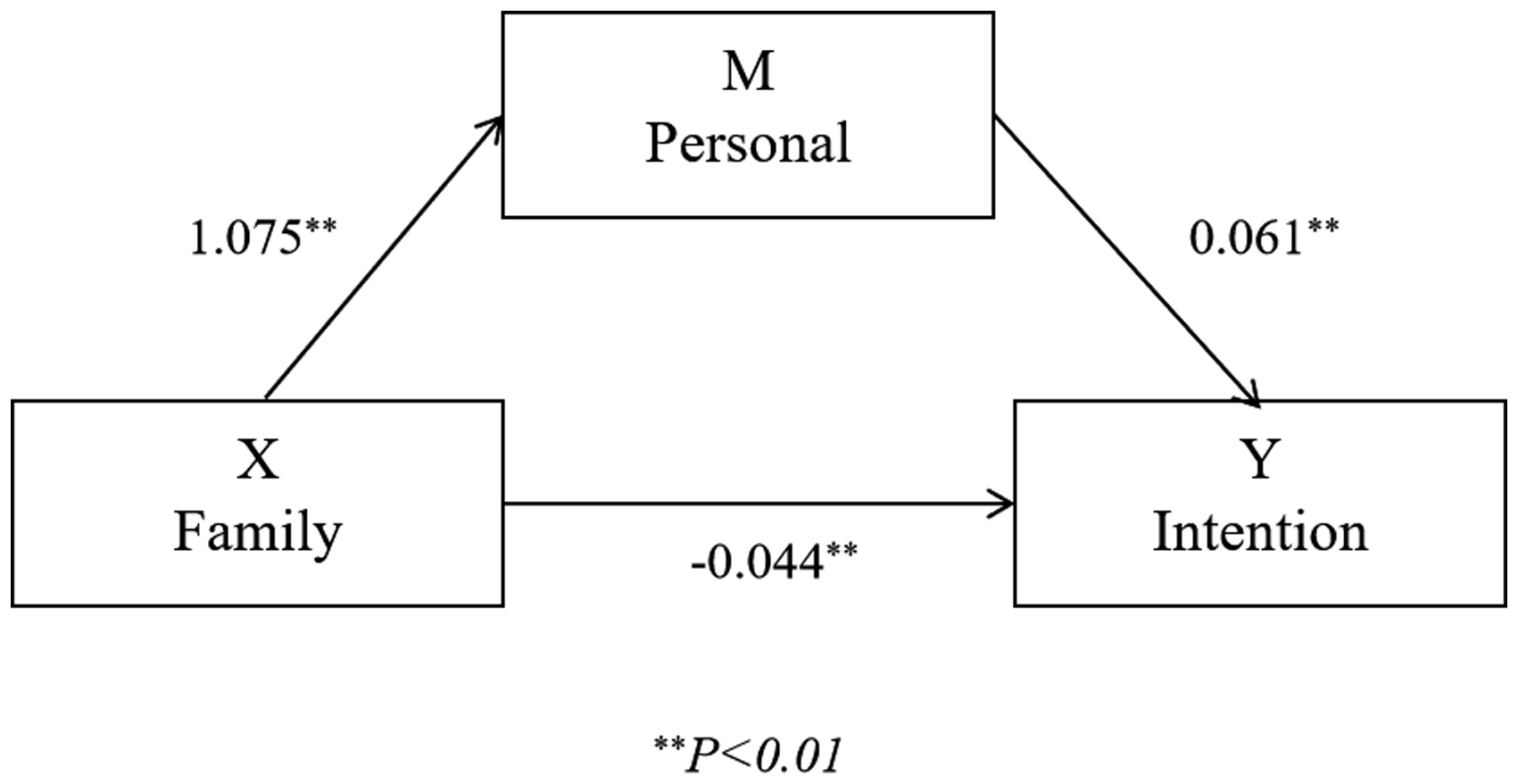
Mediation model pathway.

Residual diagnostics were also conducted for the ordinary least squares linear regression (*X* → *M*) in the first-stage model (Figure 6). Histograms of the standardized residuals and normal P–P plots suggested approximate normality, and residual-versus-fitted plots supported homogeneity of variance. Given the large sample size of *N* = 1,537, minor departures from normality would not undermine parameter estimation according to the central limit theorem. The *X*-to-*M* regression achieved good overall fit (*F* = 1,366.65, *p* < 0.001, *R*^2^ = 0.471).

**Figure 6:**
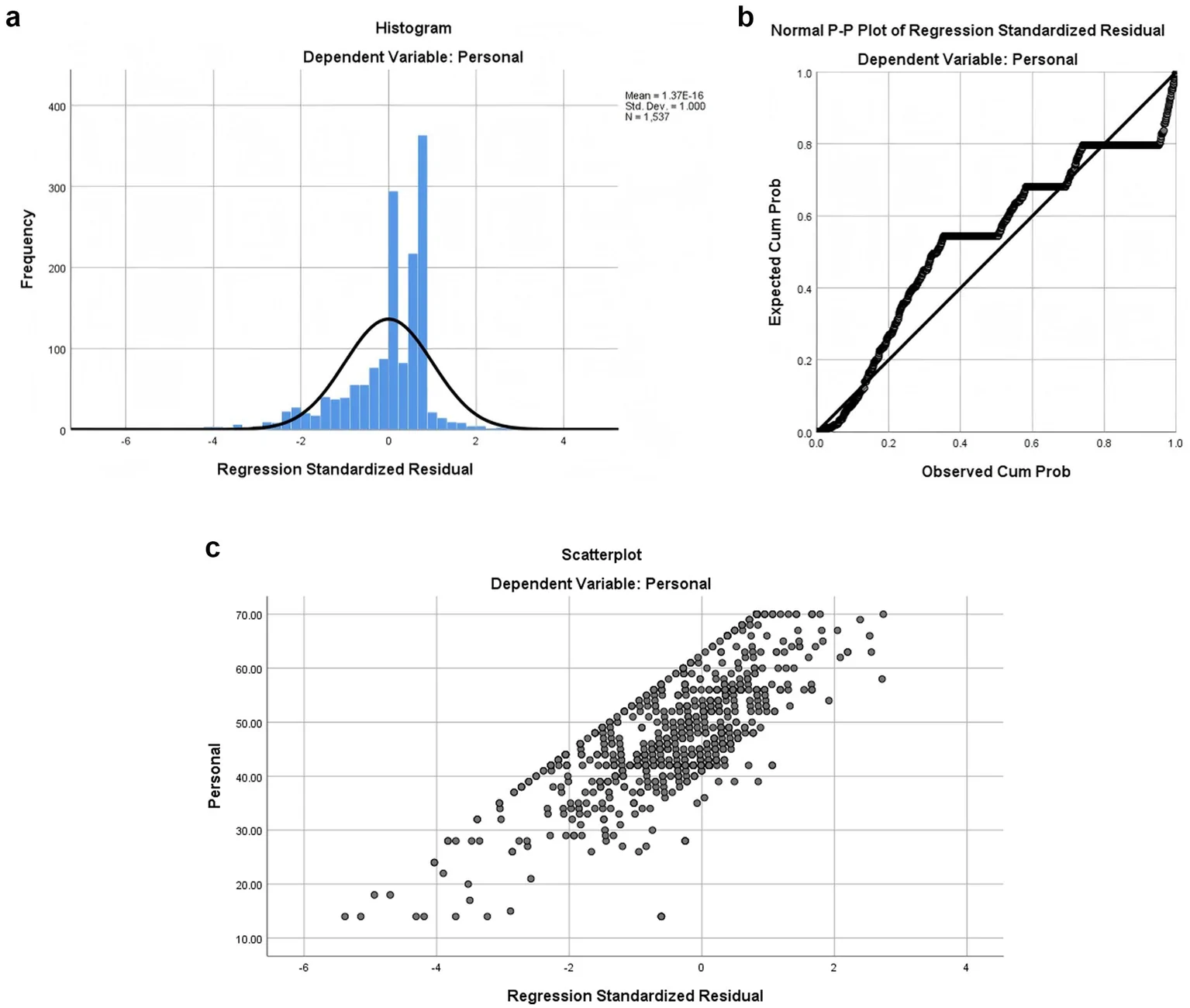
**(a)** Histogram of standardized residuals for linear regression; **(b)** Normal P–P plot of standardized regression residuals; **(c)** Scatter plot for homoscedasticity test.

## Discussion

Our findings indicate that 53.4% of vocational nursing students reported a willingness to work in geriatric care—substantially higher than the 20–30% reported in earlier Chinese research (8), yet broadly consistent with the approximately 50% willingness rate identified in a recent global systematic review (10). Local labor market conditions within Hunan Province may partly account for this pattern. Tertiary hospitals have raised academic entry requirements and increasingly favor applicants with bachelor’s degrees or above, intensifying employment competition for vocational nursing graduates. At the same time, the rapidly expanding geriatric care sector in Hunan faces substantial workforce shortages, making geriatric care positions a viable career alternative for diploma-level nursing students (23, 24). Differences in sampling regions and measurement definitions may also contribute to this discrepancy, as earlier domestic investigations were conducted mainly in eastern China and frequently adopted narrower definitions of geriatric-care work. This proportion is broadly consistent with international evidence and suggests a modest but modifiable baseline level of interest (10). From an SCCT perspective, intention to pursue a geriatric-care career arises from dynamic, reciprocal interactions among three core theoretical components: self-efficacy-related beliefs, outcome expectations, and multi-layered contextual supports and barriers spanning family, workplace, educational, and policy domains (19, 25). This framework aligns with cross-national evidence on gerontological career choice, which underscores the central roles of individual dispositions, educational exposure, and diverse contextual conditions in shaping career intentions (26, 27). Guided by this framework, the present study further examines how factors across these multiple domains jointly shape students’ willingness to pursue careers in geriatric care.

### Interpretation of key predictive factors

Interpreting this level of willingness requires attention to both structural-contextual and psychological-individual factors. Structurally, survey data identified key perceived barriers, including inadequate salary, heavy workload, and difficulties communicating with older adults. Participants preferred roles related to health management, whereas few intended to undertake physically demanding daily care or end-of-life care. Salary expectations also exceeded the remuneration available in the local geriatric care labor market.

Educational exposure plays a prominent role in shaping career willingness. Evidence syntheses indicate that structured gerontological education—including simulation training, continuous practice placement, and sustained intergenerational communication—can improve knowledge, attitudes, and occupational intentions, particularly when reflective learning targeting implicit age bias is incorporated (28). Consistent with an SCCT-informed framework, hands-on experiential inputs are positively associated with career intention, as practical experience helps shape individuals’ occupational cognitions. Both prior caregiving experience and geriatric-nursing exposure were positively associated with willingness. Within SCCT, practical contact and relevant coursework are regarded as key experiential foundations for forming occupational cognitions that encompass self-efficacy-related beliefs and outcome expectations. These observations align with intergroup contact theory, which proposes that sufficient knowledge and positive interaction reduce ageism and increase willingness to engage with older populations (29). Accordingly, vocational nursing curricula should embed progressive, long-term geriatric practice arrangements at an early stage, allowing students to gradually build professional confidence rather than relying on isolated short-term rotations.

Family-related predictors exhibited counterintuitive patterns in this study. Based on the original scale design, the composite family factors scale was further divided into three logically coherent subdimensions: family support, family occupational evaluation, and family occupational expectation and constraints. Family support reflects tangible and emotional support from parents and grandparents. Family occupational evaluation captures household and societal perceptions of the social value and prestige of geriatric care work. Family occupational expectation and constraints covers economic conditions, parental career values, social contribution expectations, and salary demands. Single-item parental support demonstrated a strong positive association with willingness, whereas the overall Family Factors total score yielded a weak negative direct effect, with no serious multicollinearity (maximum VIF = 3.419). This paradoxical suppressor effect can be attributed to the internal structural heterogeneity of the family dimension. The scale contains both promotive resources (family support) and restrictive factors (low occupational social recognition, economic constraints, and unrealistic salary expectations). The positive and negative items offset each other at the total-score level, resulting in a suppressed overall direct association. The mediation model provided further clarification of this mechanism. The positive influences of family factors were fully transmitted through the Personal Factors mediating pathway, improving students’ occupational cognitions. After controlling for the positive indirect cognitive pathway, the remaining negative direct effect largely reflected the restrictive attributes inherent in family occupational evaluations and unrealistic family career constraints. From the SCCT perspective, supportive family resources serve as critical proximal contextual supports in shaping positive self-efficacy-related beliefs and occupational intentions. However, negative family perceptions and overly utilitarian occupational expectations function as contextual barriers, offsetting supportive effects and hindering students’ willingness to engage in geriatric care. This structural imbalance within the family dimension explains why supportive family attitudes promote students’ willingness, whereas the composite family factor total score exerts an inhibitory direct effect. This pattern aligns with recent SCCT-based empirical studies demonstrating that family-related contextual factors influence career intentions primarily through individual occupational cognitions rather than through direct pathways (30, 31).

Given its theoretical relevance as a culturally specific demographic variable in China, only-child status was included in the saturated model. However, it showed no association in either chi-square univariate analysis (*χ*^2^ = 2.147, *p* = 0.143) or the fully adjusted regression. While several domestic studies have reported associations between only-child identity and geriatric-care attitudes (32), this independent effect did not emerge in our sample.

Academic year was significant in univariate analysis but became nonsignificant after full adjustment, indicating that the crude grade-based difference was essentially attributable to accumulated geriatric learning and practical experience rather than academic year itself.

Annual household income showed a weak negative independent association with willingness. It was non-significant when treated as an unordered categorical variable in chi-square analysis (*χ*^2^ = 6.338, *p* = 0.175), yet became significant when income strata were coded as an ordered predictor in the adjusted regression. The chi-square test discards ordinal rank information and therefore has lower statistical power. Within the SCCT framework, household socioeconomic background acts as a distal contextual factor shaping students’ perceived occupational alternatives. This negative association contrasts with Western research that emphasizes job stability as an attractive attribute. A plausible contextual explanation is that, under rapid urbanization in China, occupational prestige stratification makes hospital acute-care roles more desirable than long-term care positions, and cross-national comparisons confirm that local social norms substantially shape career choices (29).

Furthermore, entrepreneurial intention stood out as a strong positive correlate. Consistent with logistic regression and XGBoost outputs, this linkage can be interpreted from the SCCT perspective, which highlights occupational cognitions including self-efficacy-related beliefs and outcome expectations. China has introduced a series of policies to develop the silver economy, providing financial support and entrepreneurship subsidies for senior care services, while vocational institutions encourage student innovation projects in geriatric sectors. Students with entrepreneurial intention recognize abundant industry opportunities and regard geriatric nursing not merely as a salaried occupation but also as a potential entrepreneurial direction. Optimistic evaluations of industry prospects translate into higher willingness to engage. Such integrated policy and educational experience may offer references for other societies facing population aging. Composite scores for Personal Factors and Workplace Factors also displayed weak but statistically significant positive associations. Consistent with SCCT, individual occupational cognitions (encompassing self-efficacy-related beliefs) jointly promote career-related willingness.

### Cross-validation of predictors using logistic regression and XGBoost

To cross-validate these correlational findings, we combined conventional binary logistic regression with grid-search-optimized XGBoost. Logistic regression yields interpretable odds ratios to quantify linear associations but has limited ability to detect non-linear patterns and variable interactions. The tuned XGBoost attained a test-set AUC of 0.8186, offering stronger predictive power and the capacity to model non-linear interactions.

The two methods yielded highly consistent top-three predictors: parental support, entrepreneurial intention, and prior caregiving experience. Annual household income acted as a weak predictor in both models, reinforcing the robustness of our conclusions. Since cross-sectional observational data impose inherent limitations, neither method alone is adequate. Integrating association-focused logistic regression and prediction-oriented XGBoost enables more comprehensive analysis.

Misalignment between salary expectations and perceived market remuneration constitutes a key barrier. Within the SCCT framework, outcome expectations serve as proximal predictors of career intentions. Even among students with positive attitudes toward geriatric nursing, expected limited financial rewards and career progression reduce their willingness to enter this field.

### Mediating mechanism of personal factors

Consistent with the core propositions of SCCT, our mediation analysis further unpacked one associative pathway linking family-related contextual factors to career willingness. The results demonstrated that the composite Personal-Factors scale functioned as a significant mediating variable. In line with SCCT theory, contextual background factors rarely shape career intention in a purely direct manner; their influences are partially conveyed through individual occupational cognitions captured by the Personal-Factors scale in the present study. The Personal-Factors dimension integrates a spectrum of individual beliefs, interests, ability perceptions and work-related expectations, which correspond to the broader cognitive-appraisal constructs (including self-efficacy-related beliefs and outcome expectations) proposed within SCCT.

### Policy and practical implications

Based on the SCCT framework and the unique predictive mechanisms verified in this study, several targeted, operable practical suggestions are proposed, rather than generalized industry consensus. First, given the suppressing effect of family factors and the critical mediating role of personal occupational cognition, vocational colleges should implement family-embedded career counseling intervention tailored to nursing students. Different from conventional classroom education, schools can carry out regular family-industry sharing sessions to correct parental prejudice and stereotyped cognition toward geriatric care work; meanwhile, setting supervised family communication assignments during geriatric nursing practicum enables students to actively transmit occupational value and development prospects to their families. This targeted school-family collaborative model can effectively convert negative family constraints into contextual support, further improving students’ geriatric care career cognition and willingness.

Second, considering the significant positive prediction of entrepreneurial intention and the prominent salary expectation mismatch in this sample, colleges and industry institutions should jointly optimize career guidance and talent incentive strategies. Career education should fully integrate the policy dividends of the silver economy, guiding students to recognize the diversified employment and entrepreneurial opportunities in the geriatric care industry, rather than simply regarding geriatric nursing as a single front-line care position. In addition, targeted vocational reminder and salary outlook guidance should be provided to narrow students’ unrealistic salary expectations, alleviate occupational outcome bias, and match students’ career cognition with the actual local geriatric care labor market.

Third, combined with the positive contribution of practical experience and geriatric nursing exposure to career intention, colleges should arrange progressive and staged geriatric nursing practice training, accumulate students’ positive intergenerational interaction experience and professional self-efficacy, and form a benign contextual support path consistent with the SCCT career formation mechanism.

### Limitations and future research

This study has several limitations that should be acknowledged. First, the sample was recruited exclusively from vocational nursing students within a single province. Accordingly, the findings may not be fully generalizable to nursing students in other regions of China with distinct educational environments, socioeconomic circumstances and cultural norms and policy contexts. Future research should adopt multi-provincial or nationwide sampling to cover a more diverse student cohort.

Second, the cross-sectional design prevents causal inference. Mediation analyzes using cross-sectional data cannot establish the temporal sequence of variables and thus cannot verify causal pathways. Longitudinal tracking or interventional studies are recommended to examine dynamic changes in these relationships amid evolving education and policy contexts.

Third, all data were collected via self-report questionnaires, which may lead to recall bias and social desirability bias. Participants might overestimate or underestimate their willingness to engage in geriatric care as well as perceived family support. Subsequent research can introduce objective indicators, such as internship assessments and actual employment outcomes, to achieve data triangulation.

Beyond addressing the above methodological limitations, multiple directions merit further exploration. Mixed-methods research integrating quantitative surveys and qualitative interviews can yield an in-depth understanding of students’ real experiences and occupational decision-making mechanisms. Cross-national comparative research, for instance between China and countries with mature geriatric nursing talent training systems, helps unpack how social values, economic incentives and institutional arrangements interact with individual factors. Moreover, experimental studies evaluating targeted educational or policy interventions, such as family-involved career guidance and enriched geriatric clinical practice, can generate more robust evidence for boosting students’ willingness to pursue geriatric care careers.

Collectively, future research should expand sampling coverage, adopt more rigorous designs and fully consider contextual characteristics to validate and extend the “family–experience–cognition” framework. Relevant evidence will provide important references for optimizing nursing education and improving aged-care workforce policies in response to rapid population aging in China.

## Conclusion

This study examined the employment intentions of vocational nursing students toward geriatric care in central China and identified several key determinants. Family support, prior caregiving experience, and personal-factor-related occupational cognitions were strong positive predictors, while structural barriers—including limited curricular exposure and perceptions of poor career prospects—significantly reduced willingness to enter the field.

By applying SCCT to the Chinese vocational-education context, our findings demonstrate that contextual and personal factors interact to shape students’ career pathways. Educational experiences and family attitudes can either reinforce or undermine students’ occupational cognitions, which in turn influence their geriatric-care employment intentions. This study provides empirical evidence for the multi-dimensional “family-experience-cognition” framework.

Although our direct empirical findings are contextual to central-China settings, this analytic framework may be informative for other provinces of China as well as for international jurisdictions grappling with workforce shortages in long-term care amid rapid population aging.

This study examined the employment intentions of vocational nursing students toward geriatric care in central China and identified several key determinants. Family support, prior caregiving experience, and personal-factor-related occupational cognitions were strong positive predictors, while structural barriers—including limited curricular exposure and perceptions of poor career prospects—significantly reduced willingness to enter the field.

By applying SCCT to the Chinese vocational education context, our findings demonstrate that contextual and personal factors interact to shape students’ career pathways. The results highlight that educational experiences and family attitudes can either reinforce or undermine students’ occupational cognitions, which in turn influence their employment intentions.

These insights have practical implications for workforce development. Policymakers and educators should strengthen geriatric content within vocational curricula, foster meaningful clinical exposure, and engage families in career guidance to counter status-driven biases. In addition, supportive career structures and visible advancement opportunities are essential to ensure that educational gains translate into long-term commitment to geriatric care.

Ultimately, this study provides empirical evidence for the “family–experience–cognition” framework and underscores the need for coordinated educational and policy interventions. Although our direct empirical findings are contextual to central China settings, this analytic framework may be informative for other provinces of China as well as for international jurisdictions grappling with workforce shortages in LTC amid rapid population aging.

## Data Availability

The raw data supporting the conclusions of this article will be made available by the authors, without undue reservation.
